# Water–Gas Shift over Pt Nanoparticles Dispersed on CeO_2_ and Gadolinium-Doped Ceria (GDC) Supports with Specific Nano-Configurations

**DOI:** 10.3390/nano14231928

**Published:** 2024-11-29

**Authors:** Athanasios Androulakis, Ersi Nikolaraki, Catherine Drosou, Kalliopi Maria Papazisi, Stella Balomenou, Dimitrios Tsiplakides, Konstantinos G. Froudas, Pantelis N. Trikalitis, Dimitrios P. Gournis, Paraskevi Panagiotopoulou, Ioannis V. Yentekakis

**Affiliations:** 1 School of Chemical and Environmental Engineering, Technical University of Crete, 73100 Chania, Greece; aandroulakis@tuc.gr (A.A.); enikolaraki@tuc.gr (E.N.); edrosou@tuc.gr (C.D.); dgournis@tuc.gr (D.P.G.); 2 Chemical Process and Energy Resources Institute, Centre for Research and Technology Hellas, 57001 Thessaloniki, Greece; papazisi@certh.gr (K.M.P.); stellab@certh.gr (S.B.); dtsiplak@chem.auth.gr (D.T.); 3 Department of Chemistry, Aristotle University of Thessaloniki, 54124 Thessaloniki, Greece; 4 Department of Chemistry, University of Crete, Voutes, 71003 Heraklion, Greece; k.froudas@uoc.gr (K.G.F.); ptrikal@uoc.gr (P.N.T.); 5 Institute of GeoEnergy, Foundation for Research and Technology-Hellas, 73100 Chania, Greece

**Keywords:** WGS, Pt/CeO_2_, Pt/GDC, nanorods structure, ceria nano-morphologies, in situ DRIFTS

## Abstract

The water–gas shift (WGS) reaction is one of the most significant reactions in hydrogen technology since it can be used directly to produce hydrogen from the reaction of CO and water; it is also a side reaction taking place in the hydrocarbon reforming processes, determining their selectivity towards H_2_ production. The development of highly active WGS catalysts, especially at temperatures below ~450 °C, where the reaction is thermodynamically favored but kinetically limited, remains a challenge. From a fundamental point of view, the reaction mechanism is still unclear. Since specific nanoshapes of CeO_2_-based supports have recently been shown to play an important role in the performance of metal nanoparticles dispersed on their surface, in this study, a comparative study of the WGS is conducted on Pt nanoparticles dispersed (with low loading, 0.5 wt.% Pt) on CeO_2_ and gadolinium-doped ceria (GDC) supports of different nano-morphologies, i.e., nanorods (NRs) and irregularly faceted particle (IRFP) CeO_2_ and GDC, produced by employing hydrothermal and (co-)precipitation synthesis methods, respectively. The results showed that the support’s shape strongly affected its physicochemical properties and in turn the WGS performance of the dispersed Pt nanoparticles. Nanorod-shaped CeO_2,NRs_ and GDC_NRs_ supports presented a higher specific surface area, lower primary crystallite size and enhanced reducibility at lower temperatures compared to the corresponding irregular faceted CeO_2,IRFP_ and GDC_IRFP_ supports, leading to up to 5-fold higher WGS activity of the Pt particles supported on them. The Pt/GDC_NRs_ catalyst outperformed all other catalysts and exhibited excellent time-on-stream (TOS) stability. A variety of techniques, namely N_2_ physical adsorption–desorption (the BET method), scanning and transmission electron microscopies (SEM and TEM), powder X-ray diffraction (PXRD) and hydrogen temperature programmed reduction (H_2_-TPR), were used to identify the texture, structure, morphology and other physical properties of the materials, which together with the in situ diffuse reflectance Fourier transform infrared spectroscopy (DRIFTS) and detailed kinetic studies helped to decipher their catalytic behavior. The enhanced metal–support interactions of Pt nanoparticles with the nanorod-shaped CeO_2,NRs_ and GDC_NRs_ supports due to the creation of more active sites at the metal–support interface, leading to significantly improved reducibility of these catalysts, were concluded to be the critical factor for their superior WGS activity. Both the redox and associative reaction mechanisms proposed for WGS in the literature were found to contribute to the reaction pathway.

## 1. Introduction

The water–gas shift reaction (1) has been proposed as an effective approach for the production of hydrogen and the removal of the by-product, carbon monoxide (CO), required in several practical applications, including ammonia synthesis, Fischer–Tropsch synthesis and fuel cells [1,2,3].

(1) $$\mathrm{CO}+H_{2}O\;↔\;{\mathrm{CO}}_{2}+H_{2}\;\;{\Delta H}_{298Κ}^{0}=-41.1\;kJ/mol$$

Specifically, the WGS reaction is considered an essential processing step in the H_2_ Polymer Exchange Membrane (PEM) fuel cell technology since the elimination of CO is mandatory for the purification of the gas stream in the outlet of fuel reformers in order to avoid poisoning of the fuel cell anode [3,4,5].

It is an exothermic reaction that is controlled by thermodynamics, leading to incomplete CO conversions. As a result, the achievement of low CO concentrations is only feasible at low temperatures, whereas high reaction rates can only be accomplished at high temperatures. Thus, in industrial-scale applications, the WGS reaction is proceeded in two stages: a high-temperature (HT) stage taking place at 350–450 °C followed by a low-temperature (LT) stage operating at 180–250 °C. Fe–Cr- and Cu–Zn-based catalyst formulations have been traditionally used for the HT and LT WGS reactions, respectively [3,6]. However, several disadvantages, including catalyst pyrophoricity, particle sintering, chromium toxicity and the need for long-time activation procedures, have turned attention towards the development of alternative WGS catalysts.

Among the catalytic materials investigated so far, noble metal (e.g., Au, Pt, Pd) nanoparticles dispersed on reducible metal oxide supports, e.g., CeO_2_, TiO_2_, Fe_2_O_3_, ZrO_2_, Gd_2_O_3_-doped CeO_2_ (GDC), etc., were found to be efficient WGS catalysts, with the CeO_2_-based supports offering certain advantages mainly due to their physicochemical properties resulting in exceptionally high WGS activity and selectivity [1,3,4,5,7,8,9,10,11,12,13,14] through enhanced metal–support interactions. It is argued that the active sites for the WGS reaction are located at the interface between the metal and the support. For example, it has been reported that well-dispersed gold nanoparticles on ceria-based supports result in an increase in the active sites at the metal–support interface, which may be related to the high activity and stability for the WGS reaction [13,14]. The presence of Pt or Au on CeO_2_ surface was also found to improve the reducibility and the oxygen storage capacity of CeO_2_, favoring catalytic activity [15,16,17]. Li et al. [1] reported that the dynamic behavior of the perimeter Pt^0^−O vacancy−Ce^3+^ sites, which can be formed in the active structure, transforms under reaction conditions controlling the behavior of the adsorbed surface species, thus becoming vital for the WGS reaction. The interface of Pt/CeO_2_ with adjacent oxygen vacancies was also demonstrated by Yuan et al. [18] to serve as the active sites for the WGS reaction. According to the authors, the metal–support interactions induced by the interfacial electronic and geometric effects, as well as the abundant oxygen vacancies, were responsible for the high catalytic activity obtained. Moreover, Pt nanoparticles encapsulated in CeO_2_ over-layers were found to present similar high activity with the conventional Pt/CeO_2_ catalyst for the HT WGS reaction and were also able to completely suppress the undesired CO methanation reaction [9]. It was also demonstrated that thin (<1 nm) CeO_2_ over-layers were beneficial for the WGS reaction by promoting the formation of active oxygen vacancies. On the other hand, Fu et al. [15] demonstrated that the WGS activity depends strongly on the presence of nanosized ceria particles.

Tepamatr et al. [19] reported that when Cu is supported on gadolinia-doped ceria (GDC), it exhibits higher catalytic activity compared to CeO_2_, which can be further improved by adding Re due to enhancement of Ce^4+^ reduction to Ce^3+^. The same group recently suggested that a key step in the WGS pathway is the Ce^4+^/Ce^3+^ redox cycle over Ni and Ni-Re nanoparticles dispersed on GDC [20]. According to the authors, the formation of oxygen vacancies on the ceria surface is favored when this redox cycle is facilitated, contributing to an increase in the WGS activity and stability. Moreover, an increase in ceria reducibility induced by the presence of gadolinium was observed in our previous study over doped Pt/CeO_2_ catalysts, promoting the WGS activity [7].

The interest for the development of catalyst supports with tunable crystallite shape at nanoscale (e.g., nanorods, nanocubes, nanoflowers, nanopolyhedrals, etc.) has increased during the last few years in an attempt to alter the metal–support interactions of the dispersed metal nanoparticles and, thus, the catalytic performance of several catalytic reactions, including the CO or VOCs oxidation, WGS and CO_2_ methanation reactions [21,22,23,24]. The shape effect of CeO_2_ has been intensively studied due to the wide technological applications of this material in heterogeneous catalysis [25,26]. Many researchers agree that the morphology of CeO_2_ crystals originates from the selective exposure of different crystal planes and has a strong impact on the physicochemical properties of the supported catalysts and, consequently, on the catalytic activity [22,25,26,27,28,29]. Significant to the heterogeneous catalysis material characteristics, such as the oxygen storage capacity (OSC) and oxygen vacancies of CeO_2_, the dispersion of metal particles on its surface as well as the metal–support interactions can be optimized by controlling the size and shape of ceria particles [21,30,31]. Regarding oxygen vacancies in CeO_2_-based supports, Raman spectroscopy studies clearly demonstrated their abundance in nanorod-shaped CeO_2_ crystallites compared to other nanoshapes, such as nanoparticles and nanocubes [30,31]. On the other hand, it is well known that CeO_2_ doped with other cations (e.g., Gd- or Zr-doped ceria, GDC and CZ, respectively) leads to solid solutions with significantly enhanced defect formation (oxygen vacancies), oxygen storage capacity and mobility, properties that have been found to be critical in catalysis, electrocatalysis and fuel cell technology in various ways [25,32,33,34,35,36,37,38,39]. For example, in modern designs of intermediate temperature solid oxide fuel cells (IT-SOFC) operating under internal reforming of methane or biogas, where WGS occurs as a significant side reaction, the use of GDC as a solid electrolyte or anode electrode is advantageous [37,38].

Regarding the effect of CeO_2_ shape on the WGS activity, it has been reported that Pt supported on cube-shaped CeO_2_ exhibits higher dispersion and, thus, higher catalytic activity compared to rod-shaped CeO_2_ [40]. Contrary to this, Torrente-Murciano et al. [28] demonstrated that Pt supported on CeO_2_ nanorods is more active compared to CeO_2_ nanocubes or nanoparticles, leading to higher H_2_ yields and lower selectivities of the side product CH_4_. This was attributed to the selective exposure of the (110) crystal plane of ceria nanorods and the weak interactions between Pt and ceria nanocubes. A high WGS activity in combination with a suppression of CH_4_ selectivity was also demonstrated over Ni catalysts supported on ceria nanorods [41]. The controversy in the literature about which nano-configuration of CeO_2_ as a catalyst support is better in WGS reactions continues for other active phases. Yuan et al. [14] found that Au dispersed on mesoporous CeO_2_ outperformed Au dispersed on CeO_2_ nanorods due to the better dispersion of metal and the number of active sites at the metal–support interface on the former catalyst. Contrary to this, Cifuentes et al. [29] synthesized Au–Cu catalysts dispersed on CeO_2_ nanorods, nanopolyhedra and nanocubes and showed the performance superiority of Au–Cu/CeO_2_ nanopolyhedra that was attributed to the high surface area and the presence of (111) and (100) lattice planes. Similarly, Ren et al. [27] showed that Cu supported on CeO_2_ nanooctahedrons was better in WGS reaction than Cu supported on CeO_2_ nanorods and CeO_2_ nanotubes due to the higher Cu dispersion, the larger amount of active moderate CuO_x_ and the enhancement of Cu–support interactions.

Based on the above discussion, it can be said that although in most cases the higher WGS activity was observed for the ceria structure characterized by higher oxygen vacancies, stronger metal-support interactions and/or higher metal dispersion, the results reported so far seem to vary with respect to the type of ceria structure that gathers these characteristics. This may be due to the different methods used for ceria synthesis and/or the nature of metal that is dispersed on the ceria surface.

As described above, different nanoshapes of CeO_2_ have been widely prepared and studied in a variety of reactions. In contrast, both the preparation and the performance of catalysts based on the different nano-configurations of mixed ceria oxides, e.g., Gd_2_O_3_-CeO_2_ (gadolinia-doped ceria, GDC), are scarce in the literature. To the best of our knowledge, nano-configurated GDC supports have not been investigated so far for the WGS reaction; only a few studies concerning solid oxide fuel cell (SOFC) anode materials can be found in the literature [42,43,44].

In the present study, the catalytic performance for the WGS reaction of low-loading (0.5 wt.%) Pt catalysts supported on CeO_2_ and GDC carriers, synthesized employing two different methods leading to materials with different nano-morphologies, was investigated for the first time on GDC-based catalysts. The effect of the support’s shape and the concomitant physicochemical properties on the WGS activity and stability is discussed. The superior activity among all catalysts studied was obtained over Pt/GDC nanorods (Pt/GDC_NRs_), which also presented excellent TOS stability. In situ DRIFTS was also carried out in order to identify the active sites and reaction intermediates involved in the reaction pathway.

## 2. Materials and Methods

### 2.1. Catalyst Preparation and Characterization

#### 2.1.1. Synthesis of CeO_2_ and GDC Carriers

For the preparation of CeO_2_ and GDC (Ce_0.9_Gd_0.1_O_2_) carriers, two different synthesis protocols were followed to form different nanostructures. Specifically, the hydrothermal and the co-precipitation synthesis methods were used, which are described in detail below.

- *Hydrothermal synthesis method:* This was used to achieve nanorod-like nanostructures of CeO_2_ and GDC carriers. For CeO_2_ nanorod production, two solutions were prepared at first, the solution A by dissolving 2.5 mols of NaOH in 160 mL of deionized water (15.6 M) and the solution B by dissolving 23 mmols of Ce(NO_3_)_3_∙6H_2_O (ACROS, 99.5%) in 40 mL of deionized water (0.58 M). Then, solutions A and B were mixed at ambient temperature and under continuous magnetic stirring for 1 h and were finally placed in a Teflon container and heated at 100 °C for 25 h. The resulting mixture was subjected to centrifugation and several washes with deionized water and ethanol to bring the pH to 7 and to remove aggregates. Finally, the resulting paste was dried overnight at 100 °C and then calcinated at 750 °C for 2h (5 °C/min). Exactly the same preparation protocol was followed to produce GDC nanorods using, in this case, solution B containing precursor salts Ce(NO_3_)_3_∙6H_2_O (ACROS, 99.5%) and Gd(NO_3_)∙6H_2_O (ACROS, 99.9%) with a mol ratio of Ce/Gd = 0.9 in order to lead to the mixed oxide Ce_0.9_Gd_0.1_O_2_.
- *(Co-)precipitation method:* The CeO_2_ and GDC carriers were also synthesized by using the traditional (co-)precipitation method as follows: For obtaining precipitated CeO_2_, an aqueous solution of Ce(NO_3_)_3_∙6H_2_O (ACROS, 99.5%), 0.5 M, was prepared under continuous stirring at room temperature for 3 h. The pH value of the mixture was adjusted to 10 by dropwise addition of ammonia solution, 1M. A yellowish “emulsion” was formed, which was filtered, dried overnight at 100 °C, and finally calcined at 750 °C for 2h (5 °C/min). Following the same procedure, but using precursor salt solutions of Ce(NO_3_)_3_∙6H_2_O (ACROS, 99.5%) and Gd(NO_3_)∙6H_2_O (ACROS, 99.9%) with a mol ratio of Ce/Gd = 0.9, the GDC precipitated carrier was also prepared.

#### 2.1.2. Synthesis of Supported Pt Catalysts

Supported platinum (0.5 wt.%) catalysts were prepared following the wet impregnation method, according to which an appropriate amount of the aforementioned carriers were added to an aqueous solution of (NH_3_)_2_Pt(NO_2_)_2_ while stirring at room temperature. After being stirred at room temperature for 20 min, the suspension formed was stepwise heated at 80 °C under continuous stirring, where it remained until evaporation of water. The as-obtained solid was dried overnight and then reduced at 300 °C in H_2_ flow (60 cm^3^/min) for 2 h.

#### 2.1.3. Catalyst Characterization

The morphology of the CeO_2_ and GDC supports synthesized through the two aforementioned methods was examined via transmission electron microscopy (TEM) studies using a JEOL JEM-2011-HR high resolution transmission electron microscope (JEOL, Tokyo, Japan), operating at 200 kV, with a point resolution of 0.23 nm and Cs = 1.0 mm. Prior to analysis, all samples were dissolved in ethanol in an ultrasonic bath for 30 min.

Scanning electron microscopy (SEM) images and EDS analysis were collected on a field emission JSM-IT700HR instrument (JEOL, Tokyo, Japan) equipped with a JEOL EDS detector (JEOL, Tokyo, Japan).

The specific surface area (SSA), total pore volume and average pore size diameter of both carriers and supported Pt catalysts were measured by applying the nitrogen adsorption technique at −196 °C using a Nova 2200e Quantachrome instrument (Boynton Beach, FL, USA) and following the procedures described in detail elsewhere [45]. The SSA was estimated using the Brunauer–Emmett–Teller (BET) method, whereas the pore volume and average pore size diameter were calculated according to the Barrett–Joyner–Halenda (BJH) model.

The crystal structure of the support was determined by employing X-ray diffraction (XRD) experiments using a BrukerAXS D8 advance diffractometer equipped with LynxEye detector (Karlsruhe, Germany) with Ni-filter and Cu Kα radiation (35 kV, 35 mA). Measurements were conducted by scanning the samples from 4 to 70° using a scanning rate of 0.5 °/min. The identification of the crystalline phase of metal oxides was accomplished by comparing the X-ray diffractograms obtained with those provided by the Crystallography Open Database (COD).

A Quantachrome/ChemBet Pulsar TPR/TPD instrument (Boynton Beach, FL, USA) equipped with an Omnistar/Pfeiffer Vacuum mass spectrometer (MS) (Aßlar, Germany) at the exit of the U-type quartz sample chamber of the instrument was used for the conduction of hydrogen temperature-programmed reduction (H_2_-TPR) experiments. The samples (100 mg) were initially oxidized at 450 °C with a flowing 20% O_2_/He mixture for 2 h. Then, the temperature was decreased to 25 °C under He flow, followed by switching the feed composition from He to 5%H_2_/He. After 5 min at these flow conditions, the TPR experiment was initiated by a linear increase in the temperature (*β* = 10 °C/min) up to 670 °C. The transient MS signal at *m/z* = 2 (H_2_) was continuously recorded by the mass spectrometer, whereas its response was calibrated based on the 5 %H_2_/He mixture used (flow rate: 15 cm^3^/min).

### 2.2. Catalytic Performance Tests

Catalytic performance experiments were conducted in a 40 cm-long quartz tubular fixed-bed reactor having an inner diameter of 4 mm and an expanded 2 cm-long section in the middle, where the catalyst was placed. The reactor was heated by an electric furnace, while the catalyst temperature was measured and controlled via a K-type thermocouple placed in the middle of the catalyst bed. Gaseous reactants were supplied from high-pressure gas cylinders, and their flow and composition were controlled by means of mass flow meters (Brooks Instrument^®^, Hatfield, PA, USA).

Steam was supplied to the reactor by an HPLC pump (LD Class Pump, TELEDYNE SSI), followed by a vaporizer maintained at 180 °C. The entire stainless steel tubing of the apparatus up to the reactor inlet was kept heated at 180 °C, in order to avoid steam condensation. The outlet reaction stream was directly fed to a condenser serving to condense water before entering the analysis system consisting of a gas chromatograph (Shimadzu GC-2014) operating with Ar carrier gas and equipped with a thermal conductivity detector (TCD) and two packed columns (Porapak-Q and Carboxen). The WGS experiments were performed at 1 bar pressure and in the temperature range of 250–650 °C using 100 mg of catalyst mass and a feed composition consisting of 10% H_2_ + 10% CO + 5% CO_2_ + 35% H_2_O/Ar, with a total flow rate of 150 cm^3^/min (i.e., a weight-base Gas Hourly Space Velocity (WGHSV) equal to 90,000 mL/g∙h). The feed composition used in the catalytic performance experiments was such that the H_2_/CO molar ratio is equal to unity and contains some amount of CO_2_, as is typically found in the outlet of the dry methane reformers [46,47]. Prior to catalytic activity tests, the catalyst was reduced in situ at 300 °C for 1 h using a 50% H_2_/Ar mixture, followed by heating at 500 °C in Ar, before switching to the reaction mixture. The reaction was held at these conditions for 45 min, and then the temperature was decreased stepwise (20–30 °C per step and held there for 30 min to ensure steady state), followed by analysis of the products until CO concentration became equal to that in the reactor inlet.

The conversion of CO ($X_{CO}$) was estimated according to the following equation:

(2) $$X_{CO}(\%)=\frac{{\left[CO\right]}_{in}-{\left[CO\right]}_{out}}{{\left[CO\right]}_{in}}\times 100$$

where [CO]_in_ and [CO]_out_ are the *v*/*v* concentrations (molar fractions) of CO in the inlet and outlet of the reactor, respectively.

### 2.3. In Situ DRIFTS Studies Under WGS Conditions

In situ DRIFTS experiments were performed under WGS reaction conditions using an iS20 FTIR spectrometer (Nicolet) equipped with an MCT detector operating at liquid nitrogen temperature (−196 °C), a KBr beam splitter and a DRIFTS environmental chamber with ZnSe windows. The inlet of the environmental chamber was connected with a flow system used for feeding the gas stream in the chamber where the catalyst powder was placed. Specifically, a CO flux controlled by mass-flow meter is passed through a steam saturator maintained at 60 °C, creating a CO + steam WGS reaction mixture of 1% CO + 3.5% H_2_O. All gas lines were heated at > 60 °C using heating tapes to avoid steam condensation. In a similar manner to the catalyst evaluation experiments, the catalyst was initially heated under He flow at 500 °C and then reduced in situ to 300 °C in 50% H_2_/He for 1 h. The temperature was again increased to 500 °C under He flow, followed by a background spectrum collection at this temperature. Similar background spectra were recorded at certain temperatures while cooling the catalyst to room temperature. The 1% CO + 3.5% H_2_O gas stream was then fed (30 cm^3^/min) to the DRIFTS cell, and the first spectrum was collected after 15 min on stream. Similar spectra were collected at the desired temperatures following a progressive increase of temperature up to 500 °C. Each of the obtained DRIFT spectra was an average of 64 scans and collected at a resolution of 4 cm^−1^.

## 3. Results and Discussion

### 3.1. Catalyst Characterization Results

**Figure 1:**
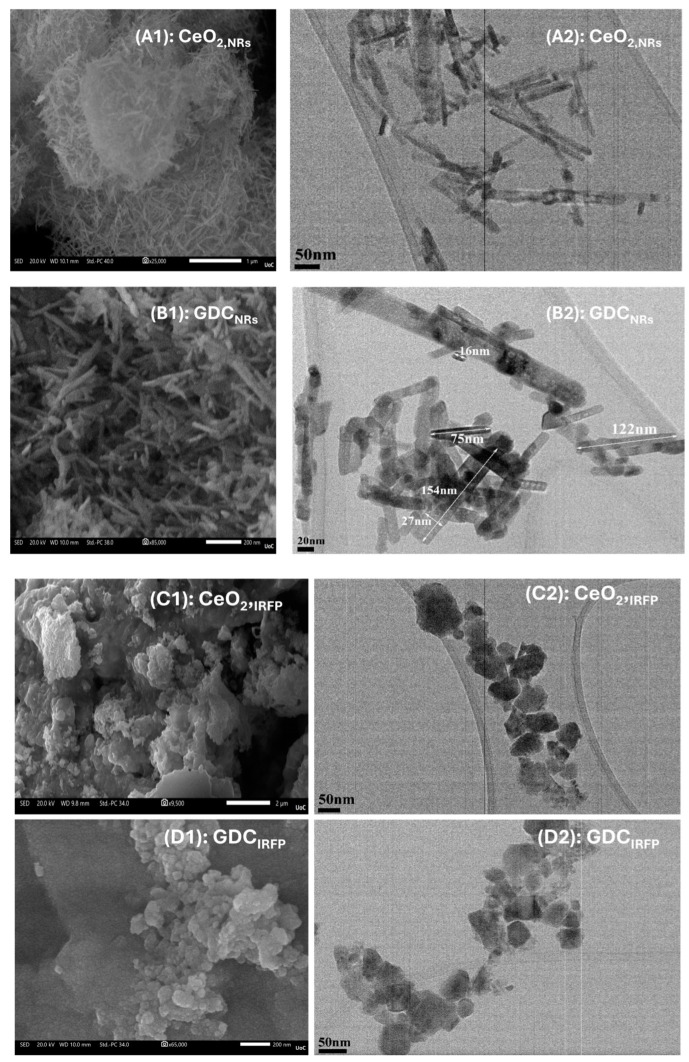
FE-SEM and HRTEM images obtained from CeO_2_ (**A**,**C**) and GDC (**B**,**D**) supports prepared by different methods leading to different nano-configurations. (**A1**,**A2**): CeO_2,NRs_, (**B1**,**B2**): GDC_NRs_, (**C1**,**C2**): CeO_2,IRFP_ and (**D1**,**D2**): GDC_IRFP_. Samples A and B were prepared with the hydrothermal method, while samples C and D with the (co)-precipitation method.

The morphology and microstructure of the CeO_2_ and GDC carriers that were synthesized were examined by FE-SEM and HRTEM, and the results obtained are shown in Figure 1. The FE-SEM and TEM results of CeO_2_ and GDC carriers prepared with the hydrothermal method are shown in A1/A2 and B1/B2 images, respectively, while those prepared with the precipitation or co-precipitation method are depicted in C1/C2 and D1/D2 images, respectively. As observed from (A) and (B), the materials synthesized with the hydrothermal method exhibit a nanorod morphology. The diameter and length of nanorods vary in both cases. Doping CeO_2_ with Gd leads to variation in the length and thickness of the resulting nanorods. In the case of precipitation or co-precipitation method for CeO_2_ and GDC, respectively, both materials show a nanograin morphology of phased-like nanoparticles of irregular shapes, as shown in Figure 1(C2,D2).

**Figure 2:**
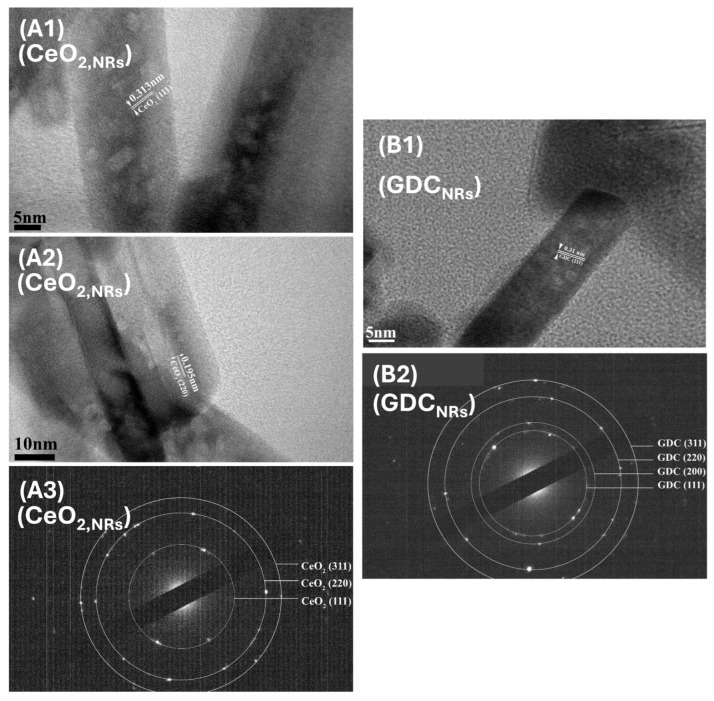
High magnification HRTEM and SAED images obtained for nanorod-shaped CeO_2,NRs_ (**A1**–**A3**) and GDC_NRs_ (**B1**,**B2**) supports.

**Figure 3:**
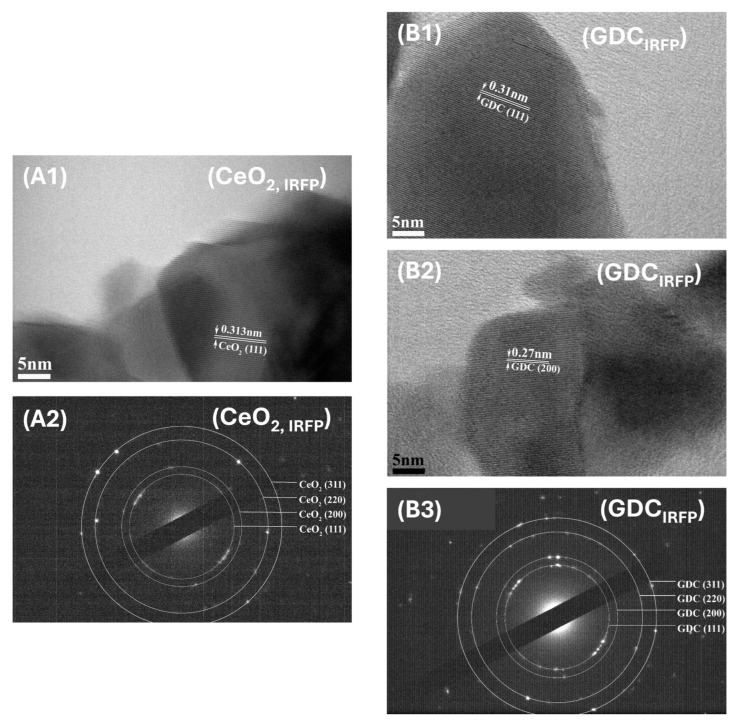
High magnification HRTEM and SAED images obtained for phased-like irregular CeO_2,IRFP_ (**A1**,**A2**) and GDC_IRFP_ (**B1**–**B3**) supports.

Higher magnification HRTEM and the corresponding electron diffraction (SAED) images of the selected areas of CeO_2_ and GDC supports (Figure 2 and Figure 3) reveal the crystalline structure of all samples, proving that both synthesis methods lead to the formation of grains in the nanoscale. The distances between the lattice fringes were measured to be 0.313, 0.270 and 0.195 nm, corresponding to the (111), (200) and (220) planes of CeO_2_ (JCPDS PDF 00-002-1306) and GDC (JCPDS PDF 01-075-0162), respectively. The CeO_2_ nanorods predominantly exposed the (111) and (220) crystal planes, while nanorods preferentially grew along (111), as shown in Figure 2(A1–A3); this agrees with similar findings in the literature [48]. The latter was also observed for GDC nanorods shown in Figure 2(B1,B2). The interplanar distance of the (111) crystal plane was measured to be 0.313 nm for both CeO_2_ nanorods (Figure 2(A1)) and nanograins (Figure 3(A1)), while for GDC nanorods and nanograins, the distance was slightly lower, 0.310 nm. This may be attributed to the gadolinium doping into the CeO_2_ lattice, which has a smaller ionic radius (Gd^+3^ (1.05 Å), Ce^+3^ (1.14 Å)) compared to Ce [49].

Notably, when Pt nanoparticles are dispersed on the above CeO_2_ and GDC supports using the wet impregnation method, their nano-configurations remain practically unaffected, as shown in the FE-SEM images of the catalysts presented in Figure S1 of the Supplementary Information File. In these FE-SEM images, Pt nanoparticles were not visible, implying a high dispersion of the low-Pt loaded (0.5 wt.%) samples, although EDS analysis of the samples showed Pt loading values between 0.42 and 0.76 wt.% Pt that were close to the nominal value of 0.5 wt.% Pt applied during the synthesis process of the catalysts. However, representative HRTEM images of our supported Pt catalysts have clearly shown Pt nanoparticles on the surface of the supports (Figure S2a). The samples exhibited uniformly dispersed spherical Pt nanoparticles with mean particle sizes lying between 0.9 and 1.1 nm regardless of the nature (CeO_2_ or GDC) and nano-morphology (nanorods or irregularly faceted particles) of the support, as shown in the corresponding Pt particle size distribution profiles (Figure S2b).

The results of nitrogen physisorption measurements are summarized in Table 1, where it is apparent that the specific surface area (SSA) of Pt catalysts dispersed on CeO_2_ or GDC support depends on the nano-morphology of the support and/or the method used for its synthesis. Specifically, the SSA of Pt supported on CeO_2_ characterized by single crystalline grains of CeO_2_ and aggregates consisting of CeO_2_ nanocrystals of irregular shapes (Pt/CeO_2_,_IRFP_) prepared by the precipitation method was found to be 20.4 m^2^/g, while that of Pt supported on CeO_2_ nanorods (Pt/CeO_2_,_NRs_) prepared using the hydrothermal method was found to be 3-fold higher (65.1 m^2^/g). The SSA results of the Pt/GDC catalysts were also qualitatively similar: the nanorod-structured Pt/GDC_NRs_ exhibited a high SSA value of 61.9 m^2^/g, whereas the SSA value for Pt/GDC_IRFP_ was about half of this at 30.5 m^2^/g.

The total pore volume of Pt/CeO_2_ catalysts significantly varied from 0.04 cm^3^/g for Pt/CeO_2,IRFP_ to 0.28 cm^3^/g for Pt/CeO_2,NRs_, whereas the corresponding average pore size diameters were equal to 7.7 and 17.0 nm, respectively (Table 1). The same trends of the total pore volume and average pore size diameter were also observed for the Pt/GDC catalysts, taking values of 0.06 cm^3^/g and 7.8 nm for Pt/GDC_IRFP_, whereas 0.26 cm^3^/g and 16.9 nm for Pt/GDC_NRs_ (Table 1).

**Figure 4:**
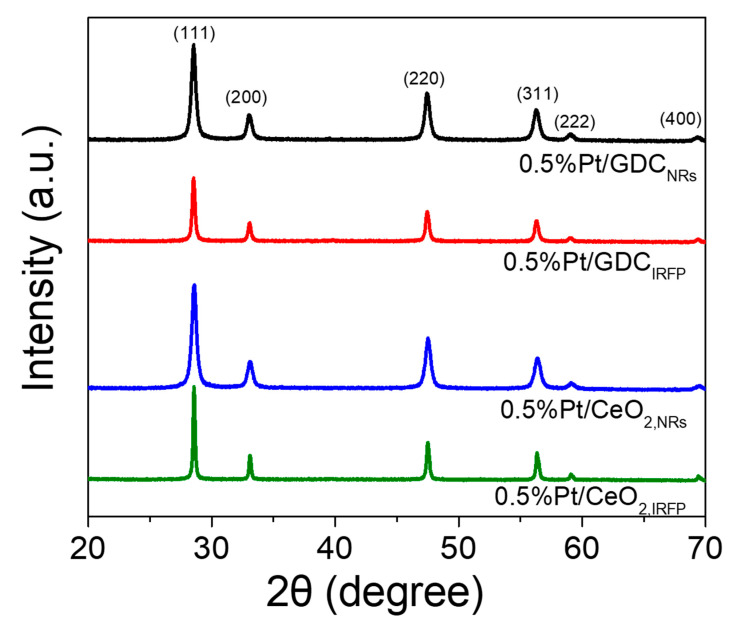
XRD patterns obtained from the reduced 0.5%Pt/CeO_2_ and 0.5%Pt/GDC catalysts with different nano-morphologies.

The XRD patterns of the Pt/CeO_2_ and Pt/GDC catalysts are presented in Figure 4. Only reflections of ceria were observable, but not of Pt, indicating a close to atomic-scale dispersion of Pt (very small nanoparticles); therefore, it is not detected in metallic form in any of the catalysts [50]. This agrees with the conclusion obtained from the analysis of the FE-SEM results.

Specifically, the diffractograms consisted of peaks located at 2θ = 28.53°, 33.09°, 47.5°, 56.29°, 59.1° and 69.37° assigned to (111), (200), (220), (311), (222) and (400) planes of cubic CeO_2_ (JCPDS Card No. 2-1306), respectively. The primary crystallite size of ceria estimated according to Scherrer’s formula based on the full width at half maximum of the CeO_2_ (111) plane was found to be 17.5 nm for Pt/CeO_2,NRs_ and 42.4 nm for Pt/CeO_2,IRFP_ (Table 1). The characteristic peaks of the cubic ceria were also discerned in the XRD patterns of Pt/GDC catalysts, with the primary crystallite sizes taking the values of 18.4 nm for Pt/GDC_NRs_ and 27.7 nm for Pt/GDC_IRFP_ (Table 1). These results implied that a strong relationship exists between the catalyst morphology/synthesis method and the textural properties, such as SSA and primary crystallite size of the support, in agreement with the results obtained by Lee et al. [40].

**Figure 5:**
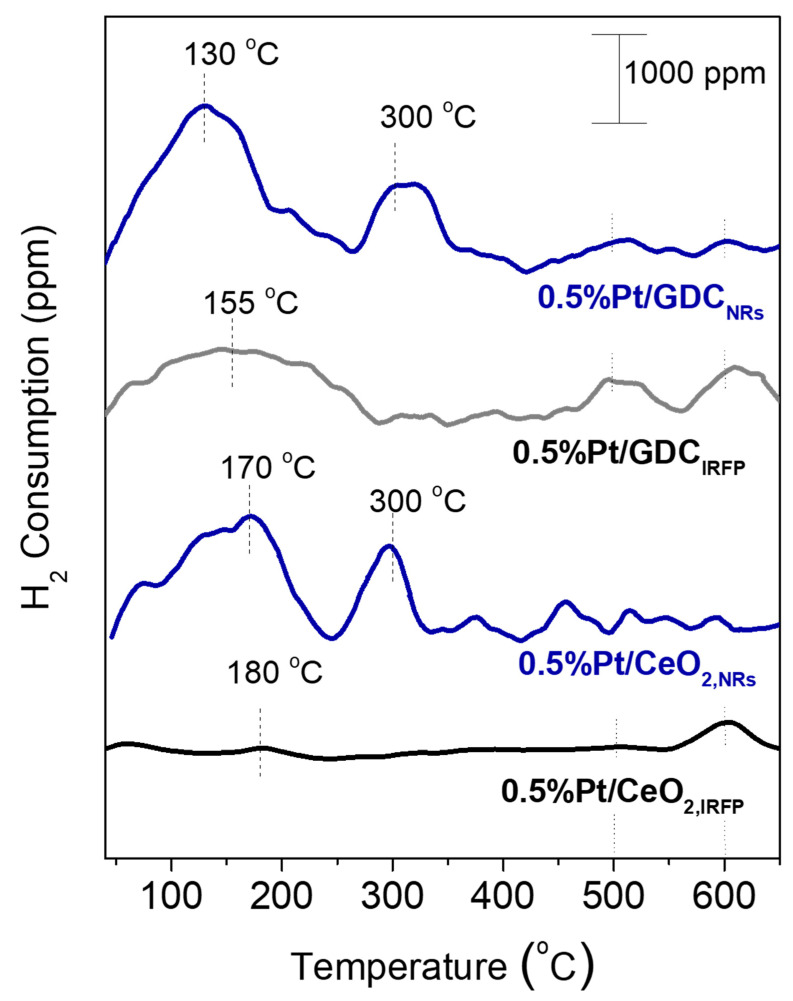
H_2_-TPR profiles obtained from the 0.5%Pt/CeO_2_ and 0.5%Pt/GDC peroxidized catalysts with nanorod (NRs) and irregularly faceted (IRFP) nanostructures.

The H_2_-TPR profiles obtained from the Pt catalysts supported on the synthesized CeO_2_ and GDC carriers are shown in Figure 5. An enhanced reducibility characterized by two hydrogen consumption features at temperatures below 400 °C, namely a low temperature peak (LT), with a maximum at about 130–170 °C, and a medium temperature (MT) feature at about 300 °C, are evident mainly for nanorod-shaped catalysts (0.5%Pt/CeO_2,NRs_ and 0.5%Pt/GDC_NRs_). The LT peak is attributed to the reduction of PtO_x_ species and/or the reduction of ceria (Ce^4+^ to Ce^3+^) in the vicinity of Pt crystallites, and the MT peak to the reduction of the support particles’ surface [7,11,17,51,52]. In contrast, the LT and MT hydrogen consumption features are rather insignificant in irregularly shaped catalysts (0.5%Pt/CeO_2,IRFP_ and 0.5%Pt/GDC_IRFP_); these catalysts show major hydrogen consumption peaks located at much higher temperatures around 500 and 600 °C.

The above TPR characteristics of the catalysts imply a more effective interaction of the Pt nanoparticles with the nanorod-shaped supports compared to that of the irregular support nano-configurations, promoting the catalyst reducibility at low temperatures, which in turn, as we shall see, plays a key role in their catalytic activity.

The amount of H_2_ consumed due to the reduction of PtO_x_ species (LT peak) and CeO_2_ or GDC crystallite surfaces (MT peak) was calculated by integrating the area below the corresponding hydrogen reduction peaks. The results showed that the amounts of H_2_ consumption as a result of PtO_x_ and/or Ce^4+^ reduction (LT and MT peaks, respectively) were both much lower for 0.5%Pt/CeO_2,IRFP_ and 0.5%Pt/GDC_IRFP_ compared to the corresponding 0.5%Pt/CeO_2,NRs_ and 0.5%Pt/GDC_NRs_ catalysts (Table 2). Overall, the best reducibility characteristics were obtained on the 0.5%Pt/GDC_NRs_ catalyst. The results of Figure 5 and Table 2 clearly indicate that the catalyst’s reducibility depends both on the nature and the crystallite morphology of the support. Evidence suggests that the nanorod structure of GDC facilitates the reduction of both PtO_x_ species and the support’s surface, as also depicted by the total oxygen storage capacity (t-OSC, in μmol O_2_/g), defined as half of the total amount of H_2_ consumed over the entire temperature range of the TPR experiment (25–650 °C), calculated from the integration of all the appearing peaks in the H_2_-TPR profile (Table 2).

It should be noted that even if considering a deep oxidation of the entire Pt amount of the 0.5 wt.% loaded catalysts during the oxidation pretreatment of the TPR experiments took place, thus converting it to PtO_2_, the theoretical amount of H_2_ consumption for its reduction is estimated to be 51.3 μmol H_2_/g. A significantly lower value was typically measured by incorporating the LT peak for the catalysts examined here (except Pt/CeO_2,IRFP_). Therefore, it is reasonable to assume that the additional amount of H_2_ consumed at low temperatures is due to hydrogen spillover associated with the reduction of ceria, which is in close contact with the Pt particles, in agreement with the previous studies [28,51]. This implies that the LT peak in H_2_-TPR profiles contains contributions from the reduction of both PtO_x_ species and Ce^4+^ cations adjacent to platinum particles in a manner similar to our previous TPR studies of platinum group metal particles supported on CeO_2_-based supports [53,54,55].

### 3.2. Effect of the Nature and Morphological Characteristics of the Support on Catalytic Performance

**Figure 6:**
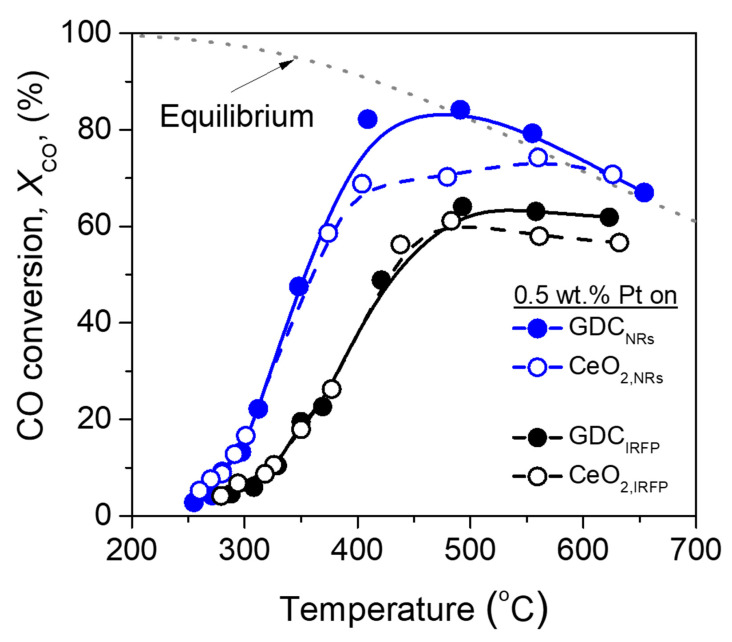
Effect of the nature and morphology of the support on the CO conversion of 0.5%Pt/CeO_2_ and 0.5%Pt/GDC catalysts for the WGS reaction. Experimental conditions: Mass of catalyst: 100 mg (particle diameter: 0.15 < d_p_ < 0.25 mm); Feed composition: 5% CO_2_ + 10% CO + 10% H_2_ + 35% H_2_O (balance Ar); Total flow rate: 150 cm^3^/min.

The two series of catalysts, Pt/CeO_2_ and Pt/GDC, using supports with different nano-morphologies, were evaluated with respect to their activity for the WGS reaction. The results obtained are presented in Figure 6, where the conversion of CO is plotted as a function of the reaction temperature. Since the WGS reaction is equilibrium limited, a detailed thermodynamic analysis was conducted using the Outokumpu HSC Chemistry^®^ 5.11 program, which can be found in the Supplementary Material (Section S1 and included Figures S3–S5); the equilibrium CO conversion anticipated by thermodynamics (equilibrium curve) for the feed composition used in the catalytic performance experiments was also plotted in Figure 6 in order to be compared with the kinetic data depicted in the figure. It is observed that Pt catalysts dispersed on nanorod-structured GDC exhibited higher CO conversions compared to those supported on CeO_2_ of the same structure at temperatures higher than 350 °C. However, both nanorod-shaped catalysts significantly outperform the irregularly shaped catalysts, Pt/GDC_IRFP_ and Pt/CeO_2,IRFP_, over the entire temperature range investigated. The best WGS behavior, obtained by Pt/GDC_NRs_, was activated above 250 °C and reached equilibrium CO conversion (X_CO_ ~85%) at about 450 °C. To the best of our knowledge, this achievement is among the best obtained so far for thermo-catalytic WGS on such low-loading (0.5 wt.%) Pt-based catalysts [56].

Traces of methane were only detected above 500 °C for Pt/GDC_IRFP_ (<0.21%) and Pt/CeO_2,IRFP_ (<0.65%), indicating that the undesirable CO/CO_2_ methanation reactions occurred to a small extent over these catalysts and were completely suppressed over Pt/GDC_NRs_ and Pt/CeO_2,NRs_ under the present experimental conditions.

**Figure 7:**
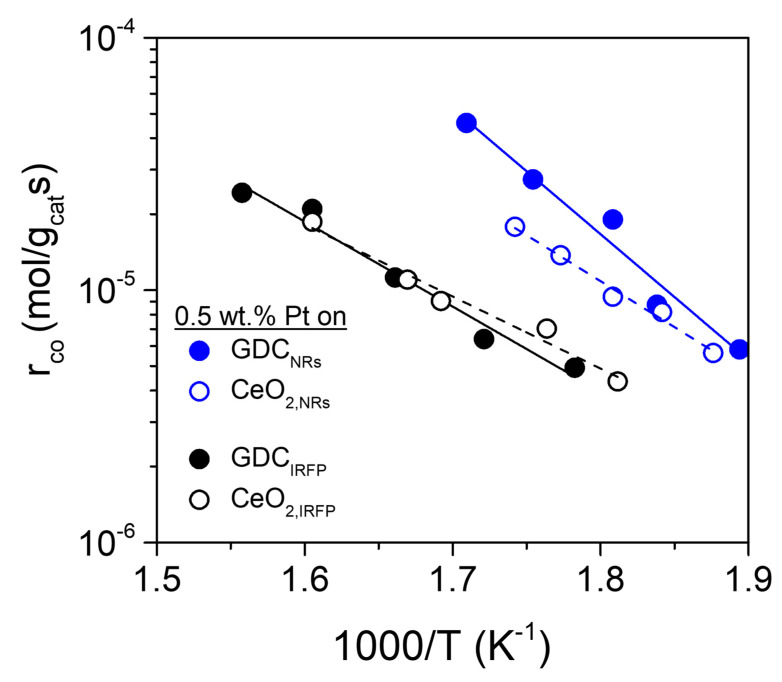
The WGS intrinsic reaction rate (Arrhenius plots) of Pt/CeO_2_ and Pt/GDC catalysts with different support nanostructures. Experimental conditions as in Figure 6. CO conversions were kept below 20%.

The intrinsic activity of the catalysts, obtained at low CO conversions (< 20%), is presented in the Arrhenius-type diagram of Figure 7. Apparently, it is strongly dependent on the type of support employed and differs up to 5-fold between the most and least active catalysts in the following order: Pt/GDC_IRFP_ ~ Pt/CeO_2,IRFP_ < Pt/CeO_2,NRs_ < Pt/GDC_NRs_. Significant changes in the apparent activation energy (*E*_a_) and the pre-exponential factor of the reaction were observed depending on the nature and structure of the support utilized, as detailed in Table 3. The so-called “compensation effect” leading to an “isokinetic effect” phenomenon, often observed in catalyst promotion studies [57,58,59,60], is evident in the present results (Figure 7 and Table 3). Indeed, the changes in the apparent activation energies between the NR- and IRFP-shaped catalysts are compensated by significant changes in the pre-exponential (entropic) Arrhenius factor, $r_{CO}^{o}$, in a similar manner to that observed in other catalytic systems, i.e., increases in *E_a_* are compensated by increases in the pre-exponential factor, thus leading to higher rates [58,59].

The results of Figure 6 and Figure 7 provide evidence that both the nature and morphology (determined by the synthesis method) of the support play a key role in the WGS activity. Regardless of whether CeO_2_ or GDC was used as support, a general trend is that the nanorod structure of the support induces significantly improved catalytic performance compared to the nanostructure of irregular shapes. This appears, at first sight, to correlate with both the higher SSA (lower primary crystallite size) and improved reducibility observed on the nanorod-structured supports (Pt/CeO_2,NRs_ and Pt/GDC_NRs_) and is in agreement with our previous studies showing that the WGS catalytic activity increases with increasing reducibility (decreasing crystallite size) of the support [7,11]. Similarly, Torrente-Murciano et al. [28] stated that the constraints of the 1D crystal structure of CeO_2_ nanorods were accountable for the superior WGS activity of the dispersed Pt catalyst, while the inferior activity of Pt supported on CeO_2_ nanocubes was related to the larger crystallite size of ceria, which hindered its re-oxidation by water. This aligns with the findings of the present study, where it was demonstrated that both CeO_2,NRs_ and GDC_NRs_ are characterized by significantly lower ceria crystallite size (17.5 and 18.4 nm, respectively) than the less active CeO_2,IRFP_ (42.4 nm) and GDC_IRFP_ (27.7 nm). However, support crystallite size should not be the only factor determining the WGS activity.

Lee et al. [40] employed two different methods (i.e., incipient wetness impregnation and strong electrostatic adsorption) in order to disperse Pt on two different ceria supports having cube and rod morphologies. They found that both the morphological properties and the catalyst particle deposition method affect the physicochemical properties of Pt/CeO_2_ catalysts and their activity for the WGS reaction as follows: Cube-shaped ceria support resulted in higher Pt dispersions and thus improved catalytic performance regardless of the Pt incorporation method. However, the latter was found to affect the number of oxygen vacancies, further improving the WGS activity. The authors concluded that high catalytic activity can be attained over catalysts characterized by well-dispersed Pt particles and a large number of oxygen vacancies. Torrente-Murciano et al. [28] suggested that the effect of metal–support interactions on the WGS activity dominates the effect of Pt particle size, which was found to be similar (ranging from 0.8 to 1.5 nm) independently of whether Pt was supported on CeO_2_ nanocubes, nanorods or nanoparticles. In our previous work, it was found that the WGS reaction is structure-insensitive, i.e., the turnover frequency (TOF) of CO conversion does not depend on metal loading, dispersion or active metal particle size for four different metal–support combinations, namely Pt/TiO_2_, Pt/CeO_2_, Pt/Al_2_O_3_ and Ru/TiO_2_ [3,8]. However, since our catalysts exhibited very similar mean Pt particle sizes (0.9–1.1 nm, Figure S2), this characteristic may be excluded from the determinants of the behavior of our catalysts regardless of whether the reaction is structure-sensitive or insensitive.

The enhanced reducibility of the nanorod-shaped catalysts (Figure 5 and Table 2) appears here to be a critical factor for their observed superior catalytic performance.

It should also be emphasized that the apparent activation energies (Table 3) were found to be lower on the irregular-shaped catalysts (Pt/CeO_2,IRFP_ and Pt/GDC_IRFP_) compared to the nanorod-shaped ones (Pt/CeO_2,NRs_ and Pt/GDC_NRs_). However, the former catalysts appear less active than the latter, indicating that the lowering of the activation energy does not contribute as expected to their activity; some other factors are more significant in this regard. Influences from mass transfer limitations (external or intraparticle diffusion) can be excluded from these factors, as all standard measures to avoid such effects were used when conducting the kinetic tests presented in Figure 7, so that they concern purely intrinsic kinetics [61]. These measures were the use of powder catalysts (grain size 150–250 μm) to avoid intraparticle diffusion limitations and sufficiently high space velocities (WGHSV = 90,000 mL/g∙h) to minimize external mass transfer limitations. In addition, the low temperatures (250–350 °C) at which these results were obtained and the low reactant conversion (*X_CO_* < 20%) are conditions that also favor the dominance of intrinsic kinetics [61]. According to chemical engineering kinetics, the absence of slope breaks on the Arrhenius lines in Figure 7 implies the absence of mass transfer constraints on these kinetic results under the conditions applied. Therefore, it is concluded that the higher surface density of active sites in the Pt/CeO_2,NRs_ and Pt/GDC_NRs_ samples, which is consistent with the observed increase of the entropic Arrhenius factor (pre-exponential factor, $r_{CO}^{o}$) in these catalysts (Table 3), is likely the critical cause contributing to their catalytic superiority, resulting from the increased reaction probability. This view is also in agreement with the more substantial interaction of Pt particles with nanorod-shaped CeO_2,NRs_ and GDC_NRs_ supports demonstrated in the H_2_-TPR results of Figure 5, which showed a larger number of undercoordinated active sites in these catalysts.

### 3.3. Time-on-Stream (TOS) and Thermal Aging Stability Tests

**Figure 8:**
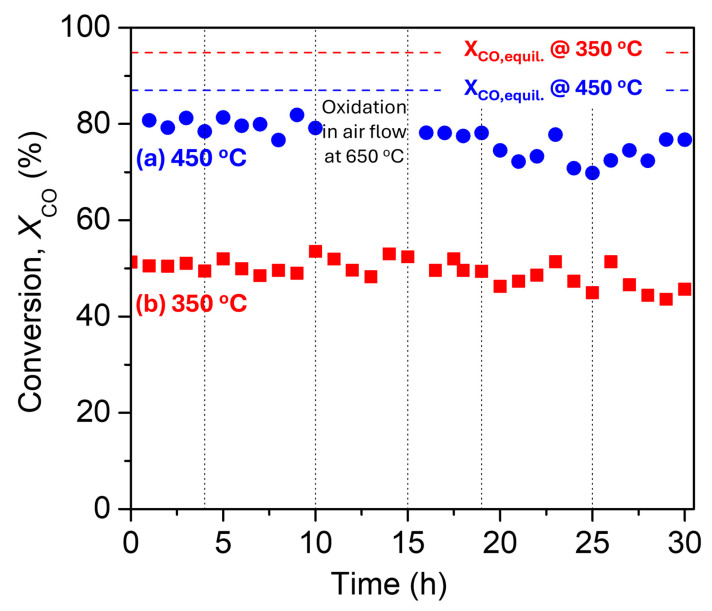
Thirty-hour TOS stability test of the best 0.5%Pt/GDC_NRs_ catalyst under conditions of WGS reaction. (**a**) at 450 °C, including an oxidative thermal aging step, and (**b**) at 350 °C without interruption. Reaction conditions: Same as in Figure 6. Dot vertical lines indicate shutting down the system overnight in He flow.

The TOS stability of Pt/GDC_NRs_, which exhibited superior performance, was investigated at two temperatures, 450 °C and 350 °C, following two different protocols, and the results obtained are presented in Figure 8. In the first protocol (Figure 8a), the catalyst was first reduced in situ at 300 °C under H_2_ flow for 1 h, followed by heating to 450 °C in He and then exposure to the reaction mixture. As can be seen, the conversion of CO remained constant and equal to ~80% for 10 h on stream. The catalyst was then subjected to a subsequent thermal aging at oxidizing conditions (i.e., exposed in air flow at 650 °C for 5 h), then reactivated by hydrogen flow at 300 °C for 1 h and re-exposed to the same WGS reaction conditions in order to evaluate its aging tolerance—such procedures are often used in practical applications as catalyst regeneration methods. It was found that *X_CO_* fluctuates between 76 and 82%, implying that Pt/GDC_NRs_ presents excellent stability even after oxidative thermal aging. A total period of 30 h was imposed in these TOS and thermal aging stability tests (Figure 8a).

The TOS stability of the Pt/GD_CNRs_ catalyst was also investigated at a lower reaction temperature, 350 °C (Figure 8b), so as to operate at a CO conversion of about 50%, considerably lower than that predicted by thermodynamics (95%). During this experimental protocol, the reactor operation was not interrupted for oxidative thermal aging, as performed in the previous one. The results showed that the catalyst exhibited excellent stability for a continuous operation of 30 h (Figure 8b).

### 3.4. In Situ DRIFTS Studies of the Catalysts Under WGS Reaction Conditions

**Figure 9:**
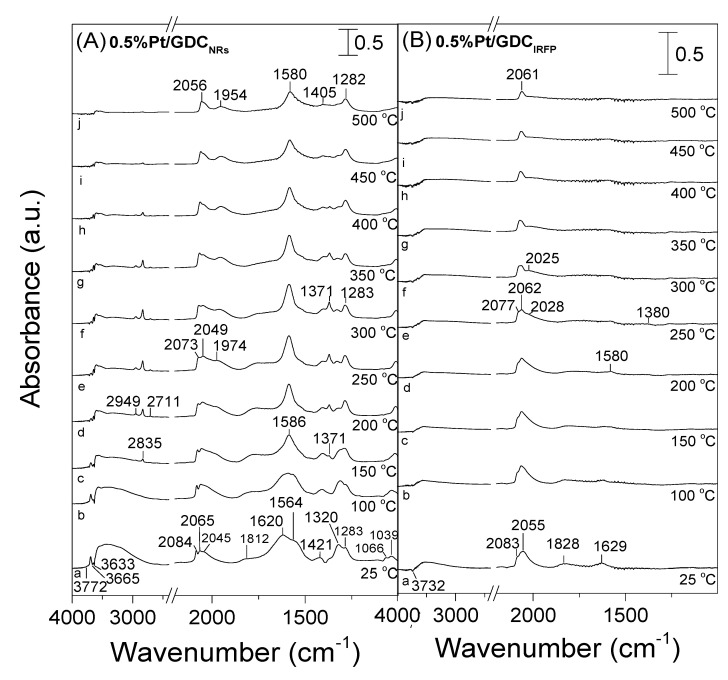
DRIFT spectra obtained from 0.5%Pt/GDC_NRs_ (**A**) and 0.5%Pt/GDC_IRFP_ (**B**) catalysts following interaction with 1% CO + 3.5% H_2_O at 25 °C and stepwise heating at 500 °C (a: 25 °C; b: 100 °C; c: 150 °C; d: 200 °C; e: 250 °C; f: 300 °C; g: 350 °C; h: 400 °C; i: 450 °C; j: 500 °C).

Operando DRIFTS experiments were carried out under WGS reaction conditions to study the effect of support morphology on the formation of reaction intermediates and the reaction pathway. The spectra recorded over the most active Pt/GDC_NRs_ catalyst following its interaction with a 1% CO + 3.5% H_2_O (in He) mixture at 25 °C and stepwise heating at 500 °C are presented in Figure 9A. The spectrum obtained at 25 °C (trace a) was characterized by two bands in the ν(CO) region located at 2084 and 2065 cm^−1^, followed by a shoulder at 2045 cm^−1^, which can be attributed to CO linearly adsorbed on Pt terrace and step-edge sites, respectively, and a low frequency band at 1812 cm^−1^, previously assigned to bridge-bonded CO at Pt terrace sites [62,63,64,65,66,67,68]. It should be noted that the shoulder at 2045 cm^−1^ may also contain contributions from carbonyls formed on platinum kink sites [62,63,64,65].

Several bands were also developed below 1700 cm^−1^, which are due to carbonate species adsorbed on GDC surface [7,34,69]. Specifically, the bands located at 1283, 1564 and 1320 cm^−1^ can be attributed to bidentate carbonate species as a result of the adsorbed CO molecule interaction with the surface oxygen species of the support [27,68,70,71]. It should be noted, however, that the formation of bicarbonate and unidentate carbonate species may also contribute to the development of the bands at 1564 and 1320 cm^−1^, respectively [71,72].

The band that appeared at 1620 cm^−1^ was previously attributed either to bicarbonate species [68] or to adsorbed steam [34] on the support surface, whereas those at 1421 and 1039 cm^−1^ were reported to be due to hydrogen carbonates [70]. The weak peak discerned at 1066 cm^−1^ corresponds to the formation of polydentate carbonate species [70,71]. Three negative bands were also observed at 3772, 3665 and 3633 cm^−1^, which characterize the OH stretching modes of three types of different surface hydroxyl groups, which are either originally present on the catalyst surface or developed as a result of catalyst interaction with steam [11,41,67,73].

A stepwise increase in temperature at 150 °C resulted in a decrease in the relative intensity of the 1620, 1564, 1320 and 1066 cm^−1^ bands, followed by the development of two new bands that appeared at 1586 and 1371 cm^−1^, which can be assigned to the OCO asymmetric and symmetric stretching vibrations of the formate species [71,73,74]. Formate formation was also responsible for the development of the C-H stretching bands at 2949, 2835 and 2711 cm^−1^, which can be clearly discerned at 200 °C and, according to previous studies, can occur under WGS conditions via interaction of the adsorbed CO species with OH groups of the support [68,73,74].

Interestingly, a new broad band can be discerned in the *ν*(CO) region located at 1974 cm^−1^ in the spectrum collected at 250 °C. This peak may also co-exist in the spectra obtained at lower temperatures but could not be discerned due to overlapping with the 2065 cm^−1^ band. Similar low vibrational frequency peaks were previously assigned to CO adsorbed on the Pt sites of exceptional electron-donating power, interacting with Ce^3+^ ions of the support [7,68,71,74,75,76]. Based on our previous works obtained over Pt/CeO_2_ and Pt/TiO_2_ catalysts, these sites located at the metal–support interface are the active sites for the WGS reaction [7,11,76].

Further increase in temperature up to 500 °C resulted in a progressive decrease in the relative population of carbonyls adsorbed on Pt sites, as well as in a decrease in the bands in the O-H stretch vibration region. It should be mentioned that the progressive decrease in the intensity of the Pt-CO bands was accompanied by their shift toward lower wavenumbers due to a decrease in the dipole–dipole coupling effect between the adsorbed CO molecules with decreasing coverage [11,67]. The intensity of the bands assigned to carbonate and formate species decreased above 350 °C, whereas the only bands discerned in the spectrum obtained at 500 °C were those located at 2056, 1954, 1580, 1405 and 1282 cm^−1^, indicating that the corresponding species are thermally more stable under the present experimental conditions.

Similar experiments were also carried out over the Pt/GDC_IRFP_ (Figure 9Β) catalyst. A comparison of the obtained spectra with those discussed above for the most active Pt/GDC_NRs_ catalyst showed the following differences: (a) The band assigned to CO species adsorbed at the metal–support interface (1974–1975 cm^−1^) was not discerned for the least active Pt/GDC_IRFP_ catalyst. This can be clearly seen in Figure S6A (Supplementary Information File), where the spectra obtained at 300 °C from the two Pt/GDC catalysts are presented in the narrow wavenumber range of 2150–1850 cm^−1^; (b) The relative population of formate species was significantly decreased with decreasing the WGS activity. Interestingly, for the least active Pt/GDC_IRFP_ catalyst, the corresponding bands below 1700 cm^−1^ were hardly discerned (Figure 9B), while those in the C-H stretching region (3050–2600 cm^−1^) were absent, indicating that the population of formate species is relatively low.

**Figure 10:**
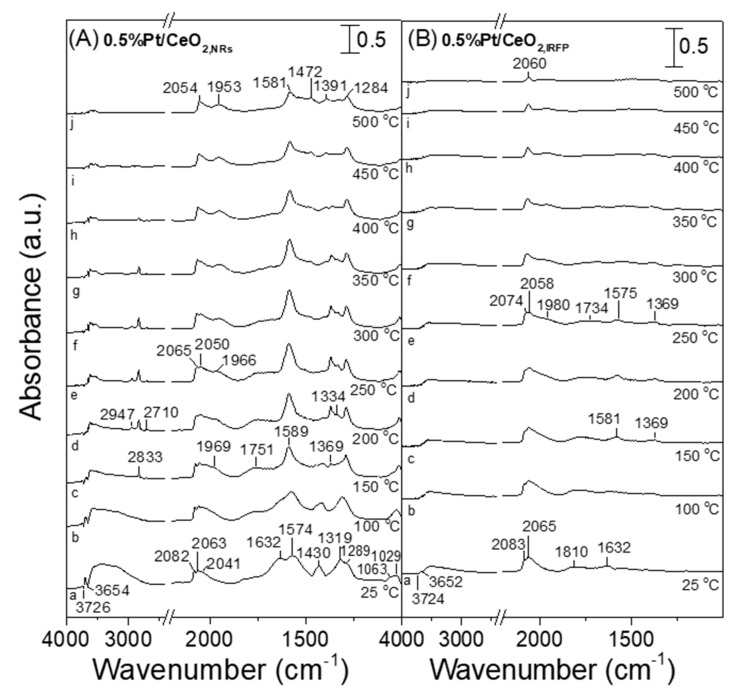
DRIFT spectra obtained from 0.5%Pt/CeO_2,NRs_ (**A**) and 0.5%Pt/CeO_2,IRFP_ (**B**) catalysts following interaction with 1% CO + 3.5% H_2_O at 25 °C and stepwise heating at 500 °C (a: 25 °C; b: 100 °C; c: 150 °C; d: 200 °C; e: 250 °C; f: 300 °C; g: 350 °C; h: 400 °C; i: 450 °C; j: 500 °C).

Qualitatively similar results were obtained over the Pt/CeO_2_ catalysts of different nanostructures (Figure 10). Particularly, the spectra collected at 25 °C following exposure of Pt/CeO_2,NRs_ catalyst to the WGS reaction 1% CO + 3.5% H_2_O (in He) mixture consisted of (a) two negative bands in the ν(OH) region (3726 and 3654 cm^−1^), associated with the consumption of surface hydroxyl groups; (b) three bands in the *ν*(CO) stretching frequency region (2082, 2063 and 2041 cm^−1^), assigned to linearly adsorbed CO on platinum terrace, step-edge and/or kink sites; and (c) several bands in the C-O stretching and deformation region, attributed to carbonate-like (1632, 1574, 1430, 1319, 1289, 1063 and 1029 cm^−1^) species adsorbed on the ceria surface. An increase in temperature to 150 °C led to the appearance of a new band at 1969 cm^−1^ due to the CO adsorbed at the periphery of platinum crystallites, which are in contact with the partially reduced ceria surface, as well as a low frequency band at 1751 cm^−1^ previously assigned to the 3-fold bridged CO species adsorbed on the metal–support interface [67,77]. The development of bands assigned to formate species (2833, 2947, 2710, 1589 and 1369 cm^−1^) was also evident above 150 °C, with their relative population presenting a maximum between 250 and 300 °C, as in the case of the Pt/GDC_NRs_ catalyst discussed above. The relative intensity of all bands decreased with further increase in temperature up to 500 °C as a result of the consumption and/or desorption of the corresponding surface species from the catalyst surface.

**Figure 11:**
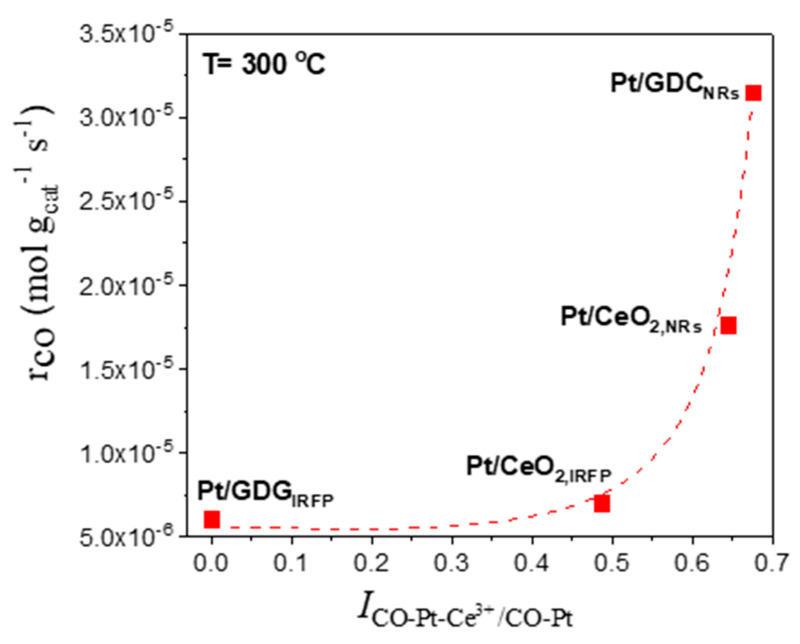
Reaction rate at 300 °C for the studied catalysts as a function of the relative intensity (*I*) of the CO-Pt-Ce^3+^ band (1960–1980 cm^−1^).

On the other hand, the population of carbonate/formate species was rather limited in the spectra obtained from the least active Pt/CeO_2,IRFP_ catalyst (Figure 10B), even at low reaction temperatures, indicating that the reactivity of the adsorbed carbonyls towards intermediate formate species was lower for this catalyst, resulting in a lower reaction rate. Moreover, the intensity of the band assigned to the CO species adsorbed on Pt−Ce^3+^ sites (1980 cm^−1^) was significantly lower compared to that observed over Pt/CeO_2,NRs_ (Figure S6B), indicating that the number of sites at the metal–support interface was lower for Pt/CeO_2,IRFP_. This further supports the above assumption that the metallic Pt sites that are in contact with Ce^3+^ ions are the active sites for the WGS reaction. In order for this to be better understood, the reaction rate measured at 300 °C was plotted as a function of the relative intensity of the CO−Pt−Ce^3+^ band normalized with respect to the CO−Pt_step sites_ band (Figure 11). It seems that the WGS reaction rate notably increases with increasing the fraction of Pt atoms at the metal–support interface relative to the amount of surface-step Pt atoms, suggesting that the sites located at the metal–support interface are the active sites for the WGS reaction.

The results of the present work are in good agreement with our previous studies over Pt/CeO_2_, Pt/TiO_2_ and alkali promoted Pt/TiO_2_ catalysts [7,11,76], where it was suggested that the appearance of the 1960–1980 cm^−1^ band upon thermal treatment indicates that the TiO_2_ or CeO_2_ support in the vicinity of Pt crystallites is partially reduced by adsorbed CO leading to the creation of sites at the metal–support interface, the number of which increases with increasing the reducibility of the support. This is in accordance with the results of Figure 5 and Table 2, where it is shown that the reducibility of both the nanorod-structured GDC and CeO_2_ was higher compared to that of the nanoparticles of irregular shapes.

**Figure 12:**
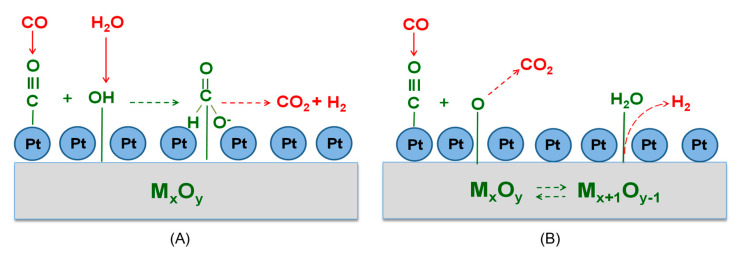
(**A**) Associative and (**B**) redox mechanistic schemes proposed for the WGS reaction.

Two general bifunctional mechanistic schemes, namely, associative and redox mechanisms, have been proposed for the WGS reaction over supported noble metal catalysts [7,11,74,76,78,79,80,81] (Figure 12). According to the associative mechanism, the CO adsorbed on the metal surface interacts with the hydroxyl groups of the support, generating an intermediate formate species that then decomposes, yielding CO_2_ and H_2_. The redox mechanism proceeds via successive reduction and reoxidation of the catalyst surface, where the adsorbed CO is oxidized by an oxygen atom of the support, producing CO_2_, followed by reoxidation of the support by adsorbed steam, releasing H_2_. An associative formate route with redox regeneration of the metal oxide used as support has also been proposed by Azzam et al. [82,83], which involves the formation of intermediate formates by hydroxyl groups, originating from the oxygen atoms of the support and not by the adsorbed steam. Based on this scheme, the oxygen atoms of the support also participate in the conversion of formates into H_2_ and CO_2_.

Regardless of which mechanism is followed in the results of the present study, the formation of oxygen vacancies on the support surface due to reduction of Ce^4+^ cations to Ce^3+^ by adsorbed CO is the primary step for the effective conversion of CO towards the reaction products. If the redox mechanism is operable, then the reduction of ceria is directly involved in the reaction pathway by oxidizing CO. On the other hand, if the WGS reaction proceeds following the associative mechanism, the reduction of ceria contributes to the creation of reactive hydroxyl groups at Ce^3+^ defect sites. The density of these OH groups is expected to increase with increasing reducibility of ceria [11,84,85]. It should be noted that the detection of bands due to formate species in the spectra presented in Figure 9 and Figure 10 at the expense of both adsorbed carbonyls and OH groups, in combination with the fact that formate species population maximizes around 300 °C and decreases at higher temperatures for the most active Pt/GDC_NRs_ and Pt/CeO_2,NRs_ catalysts, may imply that the associative mechanism (Figure 12A) dominates under the present experimental conditions. However, the redox mechanism and/or the hybrid associative formate route with redox regeneration of the support cannot be excluded.

In view of the above results, it can be suggested that the presence of partially reduced ceria particles in close contact with dispersed Pt crystallites seems to be essential for the creation of active sites at the metal–support interface, the number of which was found to progressively increase as the catalyst becomes more active (Figure 11). It may be argued that the higher activity of Pt catalysts dispersed on both GDC and CeO_2_ nanorods observed in Figure 6 could be related to the observed enhancement of the support reducibility, which favors the creation of active sites at the metal–support interface leading to an increase in the reaction rate.

## 4. Concussions

- Low-loading (0.5 wt.%) Pt-based catalysts using different CeO_2_ and GDC nano-configurations as supports (i.e., nanorods and phase-like irregular nanocrystallites resulting from the different preparation methods used) were investigated in the WGS reaction under conditions similar to those of practical applications, i.e., including CO_2_ and H_2_ in the feed (10% CO + 35% H_2_O + 5% CO_2_ + 10% H_2_).
- The WGS activity was found to depend on the nature of the support (CeO_2_ or GDC), but particularly strongly (up to 5-fold), on its nano-morphological features (irregular or nanorod-shaped crystallites). The following order of increasing activity was obtained: Pt/CeO_2,IRFP_ ≤ Pt/GDC_IRFP_ << Pt/CeO_2,NRs_ << Pt/GDC_NRs_.
- The enhanced support–metal interactions of Pt nanoparticles with the nanorod-shaped CeO_2,NRs_ and GDC_NRs_ supports compared to their irregularly structured counterparts, CeO_2,IRFP_ and GDC_IRFP_, leading to significantly improved reducibility of these samples, were found to be the critical factor responsible for their superior catalytic activity.
- In addition to the excellent WGS activity of the superior Pt/GDC_NRs_ catalyst in the present work, which is among the best recorded so far on such low-loading (0.5 wt.%) Pt-based catalysts, it also exhibits good time-on-stream and oxidative thermal aging stability.
- DRIFTS results provide evidence that the metallic Pt sites that are in contact with the Ce^3+^ ions are the active sites for the CO activation and conversion towards H_2_ and CO_2_. This was found to benefit from the enhanced reducibility of both GDC and CeO_2_ nanorods, which favors the reduction of ceria by the adsorbed CO under WGS reaction conditions involved in the reaction pathway either directly by oxidizing CO (redox mechanism) or indirectly by participating in the formation of reactive hydroxyl groups (associative mechanism).
- The present results showed that the WGS, an extremely important reaction in H_2_ production technology, both autonomously and as part of hydrocarbon reforming processes, as well as in fuel cell technology (e.g., direct biogas fuel cells), can be significantly improved in terms of its performance by interferences in the nanostructure (shape) of the catalysts. Since the materials developed as catalysts in the present work are compatible with the materials used in the design of modern intermediate temperature fuel cells, the present results provide new perspectives for both the catalytic and electrocatalytic application of these materials in several processes associated with the global effort for green energy transition.

## Figures and Tables

**Table 1:** Textural and morphological characteristics of low noble metal loading (0.5 wt.%) Pt/CeO_2_ and Pt/GDC catalysts prepared by different methods.

Catalyst	Support Preparation Method	Support Crystal Morphology	SSA (m^2^/g)	Total Pore Volume (cm^3^/g)	Average Pore Size Diameter (nm)	d_CeO2_ (nm) ^a^
Pt/CeO_2,IRFP_	Precipitation	Irregularly faceted nanoparticles (IRFP)	20.4	0.04	7.7	42.4
Pt/CeO_2,NRs_	Hydrothermal	Nanorods (NRs)	65.1	0.28	17.0	17.5
						
0.5%Pt/GDC_IRFP_	Co-Precipitation	Irregularly faceted nanoparticles (IRFP)	30.5	0.06	7.8	27.7
0.5%Pt/GDC_NRs_	Hydrothermal	Nanorods (NRs)	61.9	0.26	16.9	18.4

^a^ Primary crystallite size of CeO_2_ or GDC estimated from XRD line broadening using Scherrer’s formula.

**Table 2:** Temperature of the LT and MT peak appearance, hydrogen consumption related to the reduction of PtO_x_ species and CeO_2_ or GDC support, and t-OSC estimated based on H_2_-TPR profiles of Figure 5.

Catalyst	T_@max_ of the LT Peak (°C)	T_@max_ of the MT Peak (°C)	LT Peak	MT Peak	t-OSC (μmol O_2_/g)
			(μmol H_2_/g)		
Pt/CeO_2,IRFP_	180	-	3.6	-	25.4
Pt/CeO_2,NRs_	170	298	138.8	40.8	108.0
					
Pt/GDC_IRFP_	155	-	99.4	-	85.2
Pt/GDC_NRs_	130	313	159.1	57.6	119.6

**Table 3:** Apparent activation energy and pre-exponential factor values of the WGS reactions over the investigated 0.5%Pt/CeO_2_ and 0.5%Pt/GDC catalysts of different crystallite.

Catalyst	$\mathrm{Pre}-\mathrm{Exponential}\;\mathrm{Factor},\;r_{CO}^{o}$ (mol/g_cat ∙_s)	*E_a_* (kJ/mol)
0.5%Pt/CeO_2,IRFP_	0.68	54.7
0.5%Pt/CeO_2,NRs_	38.8	69.7
		
0.5%Pt/GDC_IRFP_	4.6	64.5
0.5%Pt/GDC_NRs_	15,658.2	95.4

## Data Availability

The original contributions presented in the study are included in the article; further inquiries can be directed to the corresponding authors.

## Supplementary Materials

The following supporting information can be downloaded at https://www.mdpi.com/article/10.3390/nano14231928/s1: Figure S1. FE-SEM images obtained from supported Pt catalysts on CeO_2_ and GDC carriers with different crystallite nano-configurations. (A): 0.5%Pt/CeO_2,NRs_, (B): 0.5%Pt/CeO_2,IRFP_, (C): 0.5%Pt/GDC_NRs_ and (D): 0.5%Pt/GDC_IRFP_; Figure S2. Representative HRTEM images (a) and the corresponding Pt particle size distributions (b) for our Pt catalysts supported on nanorods (NRs) or irregularly faceted (IRFP) support particles: 0.5%Pt/CeO_2,NRs_ (a1, b1); 0.5%Pt/CeO_2,IRFP_ (a2, b2); 0.5%Pt/GDC_IRFP_ (a3, b3). Pt nanoparticles are highlighted by cycles; Section S1: Thermodynamic aspects of the WGS reaction; Figure S3. (A): Gibbs free-energy (Δ*G*) and enthalpy (Δ*H*) changes, and (B): equilibrium constant (K) as functions of reaction temperature for the WGS reaction; Figure S4. Effect of reaction temperature on reactant and product concentrations predicted by thermodynamics for the WGS reaction using the following feed composition: 10% CO, 35% H_2_O, 10% H_2_ and 5% CO_2_ (balance Ar); Figure S5. Effect of reaction temperature on the equilibrium conversion predicted by thermodynamics for the WGS reaction using a feed composition consisting of (A) X% CO, 35% H_2_O, 10% H_2_ and 5% CO_2_ (X = 2–15) (balance Ar), (B) 10% CO, X% H_2_O, 10% H_2_ and 5% CO_2_ (X = 10–50) (balance Ar), (C) 10% CO, 35% H_2_O, X% H_2_ and 5% CO_2_ (X = 0–40) (balance Ar) and (D) 10% CO, 35% H_2_O, 10% H_2_ and X% CO_2_ (X = 0–20) (balance Ar); Figure S6: DRIFT spectra obtained at 300 °C from (A) 0.5%Pt/GDC and (B) 0.5%Pt/CeO_2_ catalysts of different nanostructures following interaction with 1% CO + 3.5% H_2_O.
