# Supplementary material for: Water–Gas Shift over Pt Nanoparticles Dispersed on CeO2 and Gadolinium-Doped Ceria (GDC) Supports with Specific Nano-Configurations
Source: Nanomaterials (Basel). 2024 Nov 29;14(23):1928. doi: 10.3390/nano14231928 (PMC11643713; doi:10.3390/nano14231928)
Supplement: Supplementary file 1 [file nanomaterials-14-01928-s001.zip › nanomaterials-3320473-supplementary.pdf]

SUPPLEMENTARY MATERIAL

Water-gas Shift over Pt Nanoparticles Dispersed on CeO_2 and Gadolinium-Doped Ceria (GDC) Supports with Specific Nano-Configurations

Athanasios Androulakis[1], Ersi Nikolaraki[1], Catherine Drosou[1], Kalliopi Maria Papazisi[2], Stella Balomenou[2], Dimitrios Tsiplakides[2,3], Konstantinos G. Froudas[4], Pantelis N. Trikalitis[4], Dimitrios P. Gournis[1,5], Paraskevi Panagiotopoulou[1,5,*], Ioannis V. Yentekakis [1,5,*]

[1] School of Chemical and Environmental Engineering, Technical University of Crete, 73100 Chania, Greece; aandroulakis@tuc.gr (A.A); enikolaraki@tuc.gr (E.N.); edrosou@tuc.gr (C.D.); dgournis@tuc.gr (D.P.G); ppanagiotopoulou@tuc.gr (P.P).

[2] Chemical Process and Energy Resources Institute, Centre for Research and Technology Hellas, 57001 Thessaloniki, Greece; papazisi@certh.gr (K.M.P.) ; stellab@certh.gr (S.B.).

[3] Department of Chemistry, Aristotle University of Thessaloniki, 54124 Thessaloniki, Greece; dtsiplak@chem.auth.gr (D.T.).

[4] Department of Chemistry, University of Crete, Voutes, 71003 Heraklion, Greece; k.froudas@uoc.gr (K.G.F.); ptrikal@uoc.gr (P.N.T.)

[5] Institute of GeoEnergy / Foundation for Research and Technology-Hellas (IG/FORTH), 73100 Chania, Greece; i.yentekakis@ig.forth.gr (I.V.Y.)

* Correspondence: igentekakis@tuc.gr or i.yentekakis@ig.forth.gr (I.V.Y.); ppanagiotopoulou@tuc.gr (P.P.)

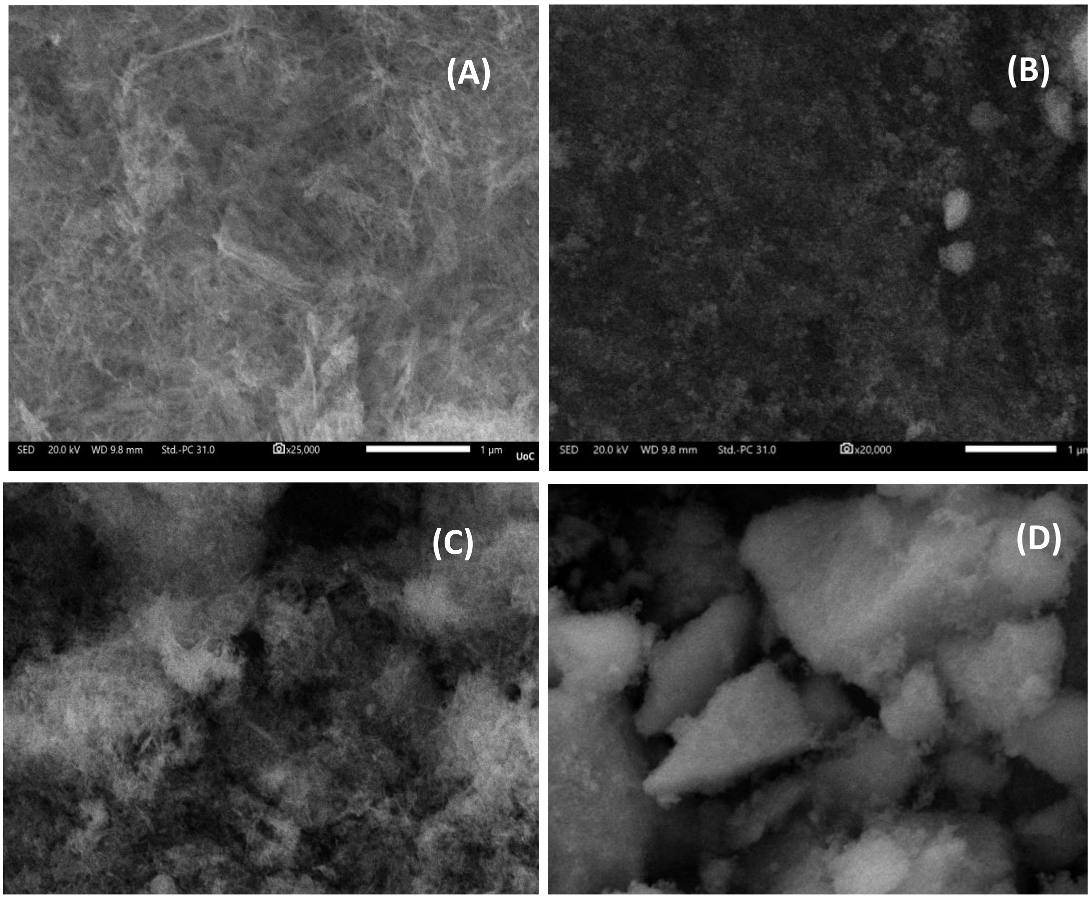

Figure S1. FE-SEM images obtained from supported Pt catalysts on CeO_2 and GDC carriers with different crystallite nanoconfigurations. (A): 0.5%Pt/$CeO_{2,NRs}$, (B): 0.5%Pt/$CeO_{2,IRFP}$, (C): 0.5%Pt/GDC_{NRs} and (D): 0.5%Pt/$GDC_{IRFP.}$

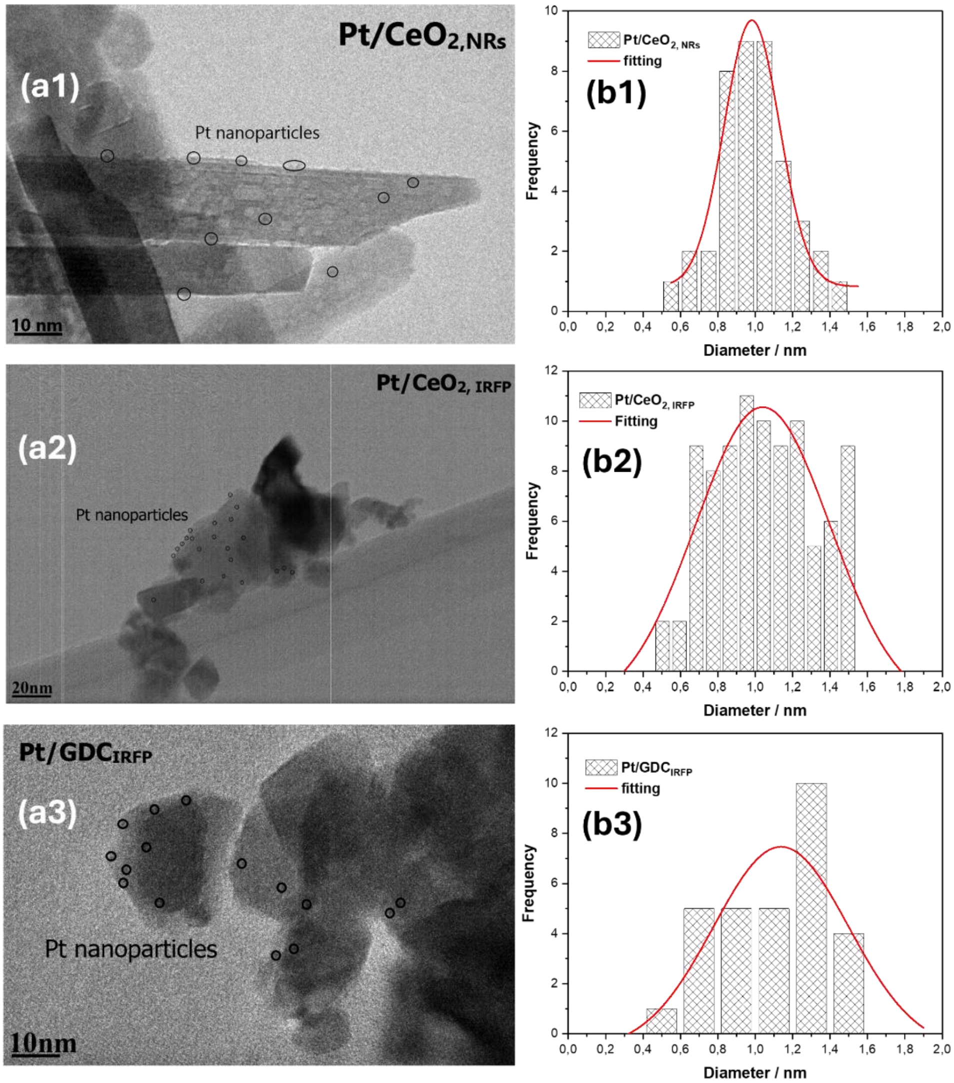

Figure S2. Representative HRTEM images (a) and the corresponding Pt particle size distributions (b) for our Pt catalysts supported on nanorods (NRs) or irregularly faceted (IRFP) support particles: 0.5%Pt/$CeO_{2,NRs}$ (a1, b1); 0.5%Pt/$CeO_{2,IRFP}$ (a2, b2); 0.5%Pt/GDC_{IRFP} (a3, b3). Pt nanoparticles are highlighted by cycles.

Section S1: Thermodynamic aspects of the WGS reaction

A detailed thermodynamic analysis of the WGS reaction is conducted herein using the Outokumpu HSC Chemistry® program. Figure S3 depicts the variations of enthalpy (ΔH_T^o) and Gibbs free-energy (ΔG_T^o) of the reaction as functions of reaction temperature. As can be seen ΔH_T^o increases from -41.138 kJ/mol to -34.879 kJ/mol with an increase in temperature from 25 to 750 °C. Throughout this temperature range the Gibbs free-energy takes negative values (Figure S3A) indicating that the reaction is thermodynamically favored in the temperature interval under consideration. The equilibrium constant (K) of the WGS reaction versus temperature is plotted in Figure S3B, and as shown it decreases with temperature.

In addition, the variation of reactant and product concentrations with temperature is calculated for the feed composition used in this study, consisting of 10% CO, 35% H_2O, 10% H_2 and 5%CO_2 (balance Ar) and results are presented in Figure S4. Along with the CO_2 and H_2 products, CH_4 can also be produced when H_2 is present in the gas stream via the CO/CO_2 hydrogenation reactions. Therefore, the possible methane formation was also considered in the thermodynamic analysis, which seems to occur below 440 °C as evidenced by the concentration of H_2 which is lower than that used in the feed. However, H_2 concentration becomes higher than its feed value of 10% above 440 °C accompanied by a decrease of CH_4 concentration, indicating that methanation reactions are eliminated at higher temperatures. It should be noted that the parallel occurrence of CO/CO_2 methanation reactions depends largely on the catalyst employed and as we saw in the present study, methanation was practically suppressed on our catalysts. Moreover, an increase of CO concentration can be observed above 450 °C implying that the reverse WGS reaction becomes operable, without, however, being predominant up to 750 °C for the examined feed composition.

The effect of each compound concentration in the gas stream on the equilibrium conversion was calculated and presented in Figure S5. Results indicate that increasing CO or H_2O concentration in the feed favors the equilibrium CO conversion, with the effect of H_2O concentration (Figure S5B) being, generally, higher compared to that of CO (Figure S5A), where no further improvement on equilibrium conversion can be achieved with increasing CO concentration from 5 to 15%. An opposite trend was observed by increasing the concentration of CO_2 or H_2 in the feed, which both inhibit the equilibrium conversion of CO (Figure S5 C and D).

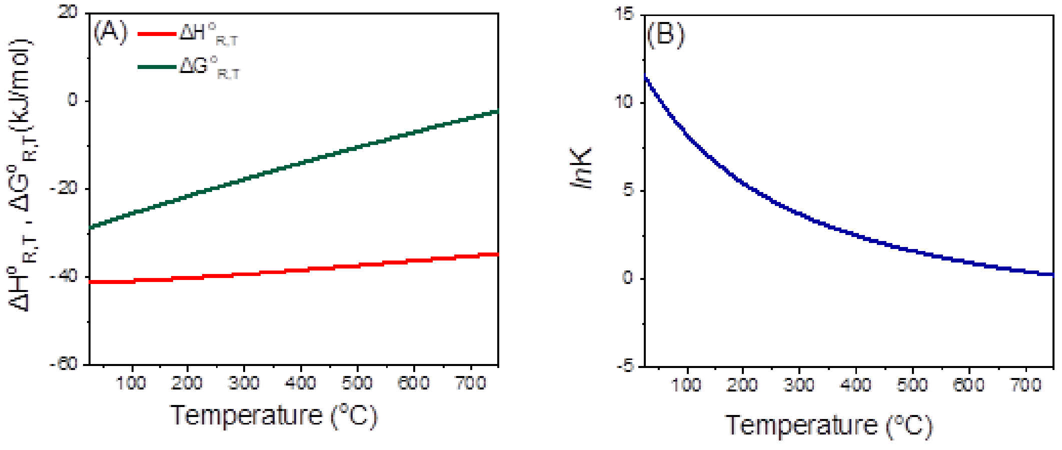

Figure S3. (A): Gibbs free-energy (ΔG) and enthalpy (ΔH) changes, and (B): equilibrium constant (K) as functions of reaction temperature for the WGS reaction.

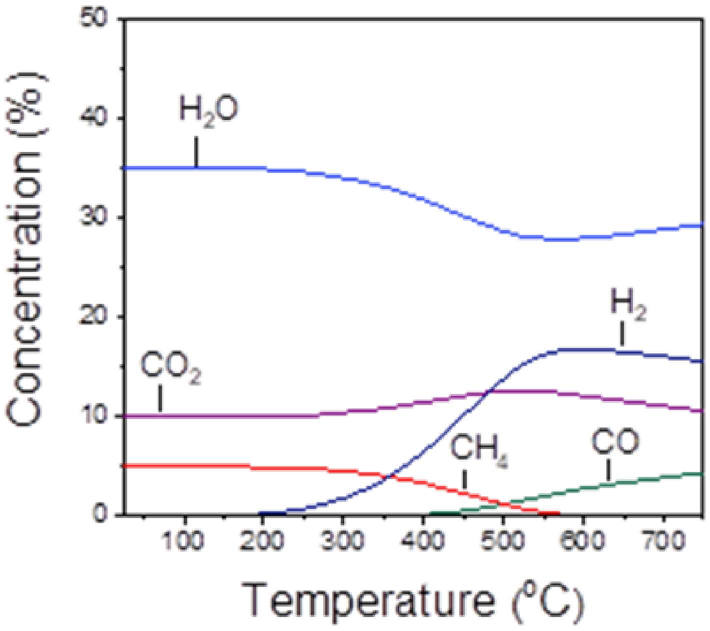

Figure S4. Effect of reaction temperature on reactants and products concentrations predicted by thermodynamics for the WGS reaction using a feed composition: 10% CO, 35% H_2O, 10% H_2 and 5% CO_2 (balance Ar).

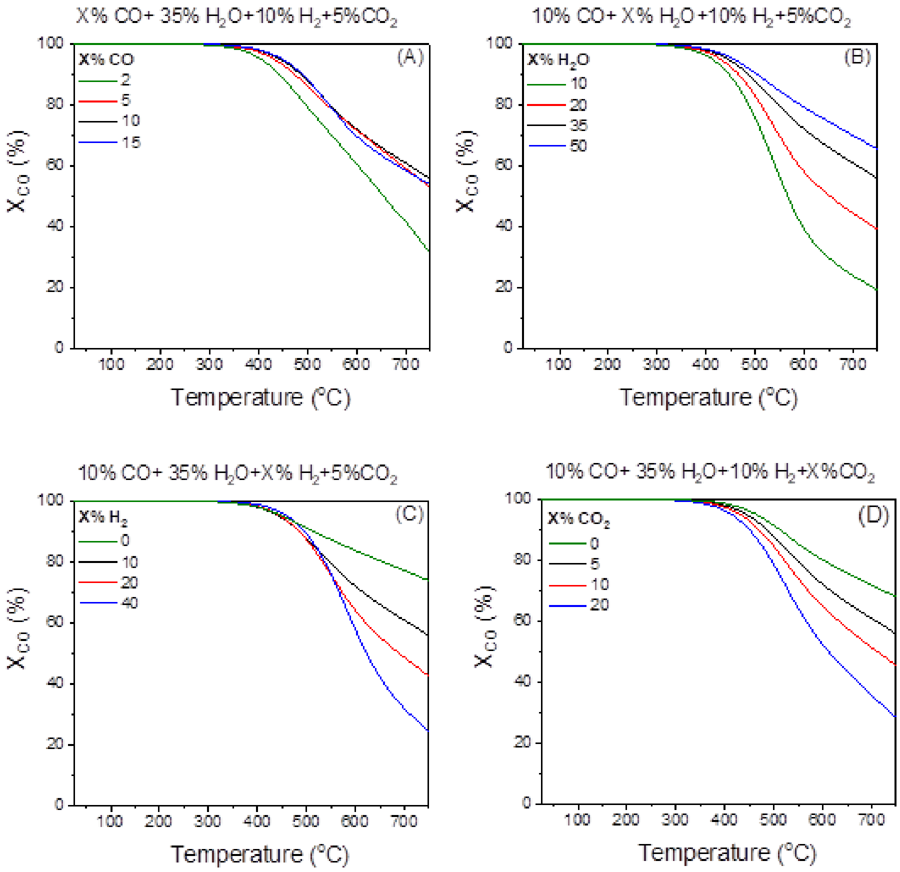

Figure S5. Effect of reaction temperature on the equilibrium conversion predicted by thermodynamics for the WGS reaction using a feed composition consisted of (A) X% CO, 35% H_2O 10% H_2 and 5% CO_2 (X = 2-15) (balance Ar), (B) 10% CO, X% H_2O, 10% H_2 and 5% CO_2 (X = 10-50) (balance Ar), (C) 10% CO, 35% H_2O X% H_2 and 5% CO_2 (X = 0-40) (balance Ar) and (D) 10% CO, 35% H_2O, 10% H_2 and X% CO_2 (X = 0-20) (balance Ar).

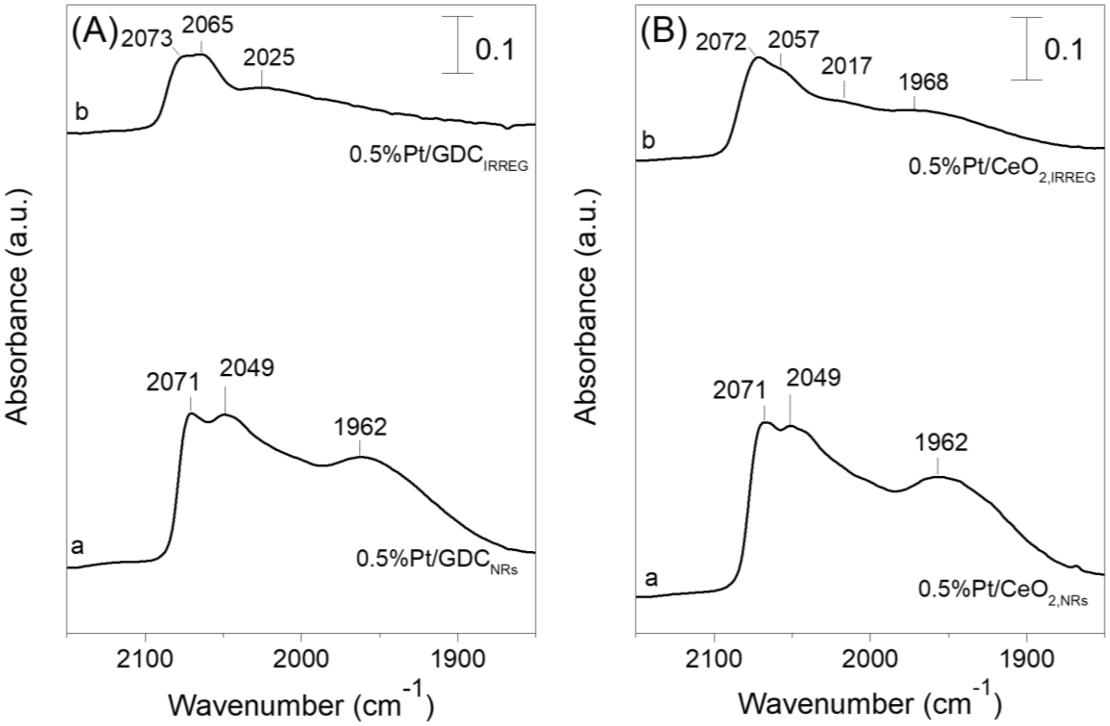

Figure S6. DRIFT spectra obtained at 300 °C from (A) 0.5%Pt/GDC and (B) 0.5%Pt/CeO_2 catalysts of different nanostructures following interaction with 1% CO + 3.5% H_2O.
